# Recent Advances in the Development of Selected Triterpenoid-Based Hybrid Molecules and Their Antimicrobial Activities: A Review

**DOI:** 10.3390/antibiotics15020185

**Published:** 2026-02-08

**Authors:** Lihle Mdleleni, Pamela Rungqu, Tobeka Naki

**Affiliations:** Department of Chemistry, Faculty of Science and Agriculture, University of Fort Hare, Private Bag X1314, Alice 5700, South Africa; 202118704@ufh.ac.za (L.M.); prungqu@ufh.ac.za (P.R.)

**Keywords:** triterpenoid hybrids, betulinic acid, oleanolic acid, ursolic acid, antibacterial activity, antifungal activity, structure–activity relationship

## Abstract

Triterpenoids are a diverse class of naturally occurring compounds with a wide range of pharmacological properties, including anticancer, anti-inflammatory, antimicrobial, and antiviral activities. Among them, ursolic acid (UA), oleanolic acid (OA), and betulinic acid (BA) have emerged as key scaffolds due to their broad therapeutic potential and structural versatility. However, the clinical application of these compounds is often limited by issues such as poor solubility, bioavailability, and selectivity. To address these challenges, research conducted between 2015 and 2025 increasingly focused on the development of triterpenoid-based hybrid molecules, in which the triterpenoid scaffolds are chemically linked to other bioactive pharmacophores. This approach aims to enhance therapeutic efficacy through synergistic action, improved pharmacokinetics, and multitarget interactions. This comprehensive review explores recent advancements in the design, synthesis, and evaluation of hybrid derivatives of selected triterpenoids, particularly UA, OA, and BA. Emphasis is placed on the structure–activity relationships (SARs), biological evaluations, and mechanisms of action of these hybrid compounds across various disease models. The review also highlights current challenges, research gaps, and future perspectives in the rational development of triterpenoid-based hybrids as potential leading candidates in drug discovery.

## 1. Introduction

Natural products have consistently served as rich sources of pharmacologically active compounds. Their complex chemical structures and bioactive profiles often exceed the capabilities of purely synthetic molecules. Among them, triterpenoids stand out as particularly promising candidates due to their low toxicity, structural versatility, and broad spectrum of pharmacological activities. In the era of rising antimicrobial resistance (AMR), the search for novel therapeutics derived from natural products has gained significant attention, as conventional antibiotics are increasingly losing their therapeutic effect [1,2,3].

**Figure 1:**
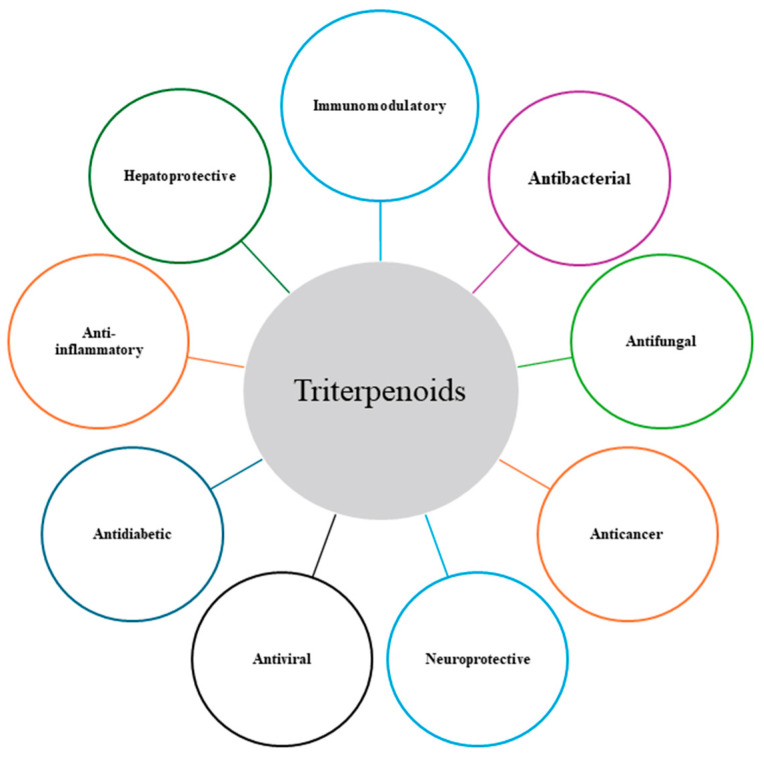
The pharmacological activities of the selected pentacyclic triterpenoids.

Triterpenoids, particularly the pentacyclic class, are a diverse group of natural compounds derived from the isoprenoid pathway and are widespread in the plant kingdom. Their unique chemical structures and broad spectrum of bioactivities have made them highly attractive scaffolds in drug discovery and development. Among them, the selected triterpenoids such as BA, OA, and UA stand out due to their well-documented pharmacological activities (Figure 1), which include anticancer, anti-inflammatory, neuroprotective, hepatoprotective, immunomodulatory, antidiabetic, antibacterial, antiviral, and antifungal effects [2,4,5,6,7,8,9,10]. These compounds are biosynthesized via the cyclization of squalene and differ structurally due to subtle modifications in their rings and functional groups, which greatly influence their biological behaviour. For instance, BA is characterised by a lupane skeleton and is commonly isolated from *Betula* species [4], while OA and UA share similar ursane and oleanane backbones, frequently found in olive leaves (*Olea europaea*), apple peels, and various herbs [2,6].

Despite their potent pharmacological activities, the clinical application of these natural molecules also presents limitations, such as poor aqueous solubility, low bioavailability, and limited target selectivity [6]. To address these limitations, recent studies have focused on designing hybrid molecules by structurally modifying BA, OA, and UA with various pharmaceutical scaffolds to enhance antibacterial and antifungal efficacy, while potentially overcoming issues of solubility and resistance [2,5,11,12]. Although pentacyclic triterpenoids are associated with a wide spectrum of pharmacological effects, antimicrobial activity was selected as the primary focus of this review due to the urgent global challenge posed by antimicrobial resistance and the diminishing effectiveness of existing antibiotics. Unlike many other biological indications, antimicrobial drug discovery faces a critical shortage of new chemical classes with novel mechanisms of action. Triterpenoid-based hybrids are particularly well suited to address this challenge, as the structural modification of these scaffolds can enhance membrane interactions, multitarget activity, and resistance-modulating effects, making them attractive candidates for next-generation antibacterial and antifungal agent development [4,5,6,7,8,9,10].

**Figure 2:**
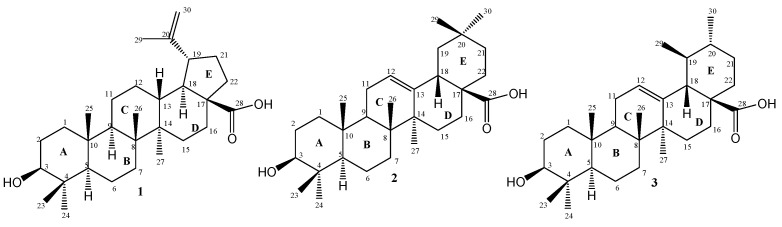
Chemical structures of the selected triterpenoid scaffolds: betulinic acid (**1**), oleanolic acid (**2**)**,** and ursolic acid (**3**). The letters A, B, C, D, and E identify carbon rings.

This review focuses on the recent progress in the development of hybrid molecules based on BA (**1**), OA (**2**), and UA (**3**) (Figure 2) synthesised through rational structural modifications. It highlights key synthetic approaches, structure–activity relationships (SARs), and biological evaluations of these hybrids. It aims to present a consolidated perspective on how such strategies can contribute to modern antimicrobial drug design [2,5,13].

The BA, OA, and UA were selected as the focus of this review due to their prominent representation in the antimicrobial triterpenoid literature and their well-established roles as versatile scaffolds for chemical derivatization. These three pentacyclic triterpenic acids are widely distributed in medicinal plants, share closely related core structures, and have been repeatedly explored in hybrid-molecule designs aiming to enhance antibacterial and antifungal activity. While other triterpenoids, such as lupeol, glycyrrhetinic acid, and asiatic acid, also exhibit diverse biological properties, their antimicrobial hybrid derivatives are comparatively less studied or fall outside the comparative scope of the present review. Accordingly, the focus on BA, OA, and UA allows for a coherent and scaffold-driven analysis of structure–activity relationships within a well-defined body of the literature [2,5,6,13,14].

## 2. Materials and Methods

This review surveyed the primary literature published between 2015 and 2025. Systematic searches were conducted using PubMed, Web of Science, Scopus and Google Scholar. Search terms combined scaffold names and design concepts, for example: “betulinic acid”, “oleanolic acid”, “ursolic acid”, “triterpenoid hybrid”, “antibacterial”, and “antifungal”. Titles and abstracts were screened for relevance, and full texts were examined when (i) the article reported synthesis of triterpenoid hybrids and (ii) antimicrobial data were provided. Chemical structures and schemes were drawn using ChemDraw (Ultra 8.0). Where possible, data were compared using the original assays and units reported by the authors to ensure consistency across studies.

## 3. Triterpenoid-Based Hybrid Molecules and Their Biological Activities

### 3.1. Betulinic Acid-Based Hybrid Molecules

#### 3.1.1. Synthetic Approaches to Betulinic Acid-Based Hybrid Molecules

Betulinic acid (BA, 3β–hydroxy–lup–20(29)–en–28–oic acid) is a pentacyclic lupane-type triterpenoid most commonly extracted from birch bark, notably from Betula species, which can produce yields up to 24–30% by dry weight [15]. However, low yields from natural sources limit scalability. To circumvent this, studies have employed chemical synthesis from betulin, a diol (3β,28–diol) largely present in birch bark (up to ~30%) [16]. Classic methods include a two-step route converting betulin to BA via oxidation of the C–28 hydroxyl to a carboxyl group using Jones oxidation or pyridinium dichromate, followed by acetyl deprotection [15,17].

Structurally, BA possesses key functional groups at C–3 (a hydroxyl) and C–28 (a carboxyl) that serve as primary sites for medicinal chemistry modifications, while C–20/C–29 and other ring carbons (C–2, C–20) are occasionally targeted for deeper functionalization [18]. The C–28 group is the most utilised handle in synthetic design. Two major types of modifications are amide formation and 1,2,3–triazole formation via Copper (I)-catalysed Azide-Alkyne Cycloaddition (CuAAC), a related reaction within the broader click-chemistry concept originally introduced by Sharpless and co-workers. Amide formation involves the coupling of the carboxyl to amines (e.g., amino acids, peptides, heterocycles) using standard reagents (EDC/HOBt, DCC, acid chlorides), which are widely used to yield amide-linked hybrids [19]. These enhance water solubility and offer platforms for linking to peptide pharmacophores or other bioactive fragments. The formation of 1,2,3–triazole via CuAAC begins with the modification of BA to an alkyne derivative at C–28, which is then reacted with azides in the presence of Cu(I) catalysis to yield triazole linkers [8]. This method is highly regioselective, tolerant of multiple functional groups, and commonly yields mono- and bis-triazole derivatives [20,21,22]. The C–3 hydroxyl is often derivatized via esterification, typically involving acylation with acid chlorides or anhydrides under DCC/DMAP conditions [4,23,24]. This process allows attachment of small molecules or prodrug moieties while enabling controlled release under physiological conditions. The glycoside formation involves attaching sugar moieties or other hydrophilic groups to increase solubility and improve targeting, particularly by exploiting glucose transporter pathways that are upregulated in cancer cells [25]. Other positions (C–2, C–20, C–29) are less frequently targeted, but certain studies have introduced substituents at C–2 or C–20/C–29, such as propargyl groups or aliphatic side chains, to explore novel binding modes or improve biological profiles [18,26].

In short, the most consistently successful strategy for improving BA is C–28 functionalization, which reliably improves antimicrobial potency and enables installation of diverse pharmacophores (amides, triazoles, dithiocarbamates). Modifications at C–3 are valuable for tuning solubility and biofilm activity, while cationic appendages (amines/guanidines) and sulfur-containing moieties often deliver the largest antifungal and Gram-positive gains. Together, these levers provide a clear, practical roadmap for converting BA into lead-like antimicrobial scaffolds.

#### 3.1.2. Antibacterial Improvements of Betulinic Acid-Based Hybrid Molecules

**Figure 3:**
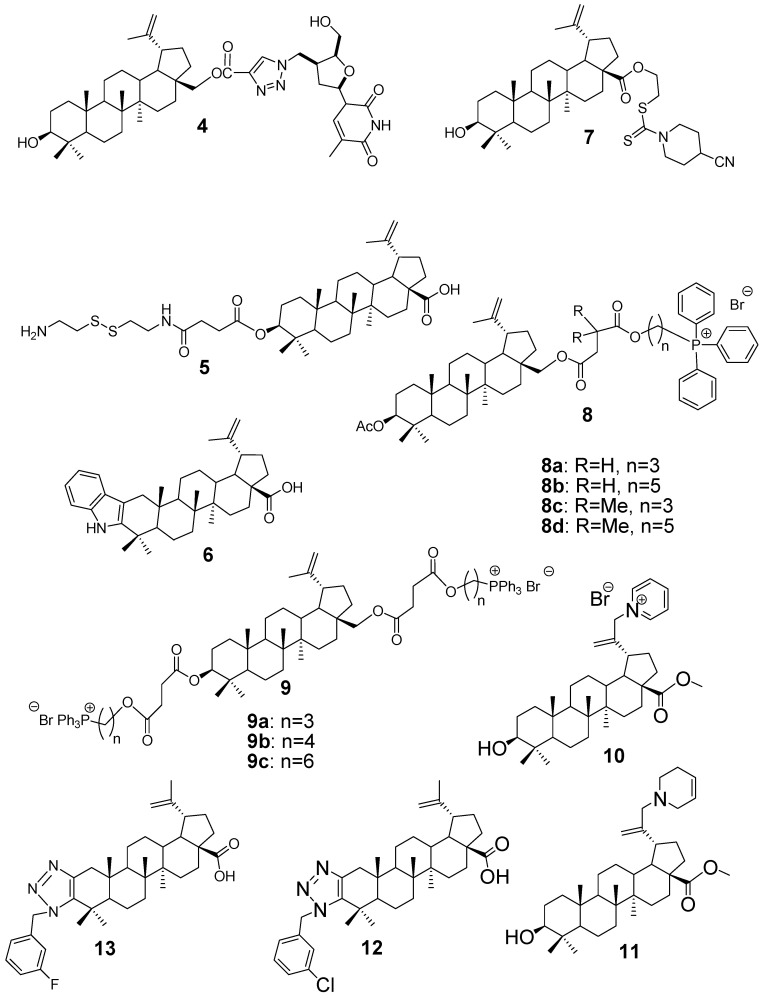
Betulinic acid-based hybrids with antibacterial activity.

Antibacterial resistance is an escalating global threat that underscores the urgency for new chemical entities with enhanced efficacy or synergistic potential. BA, a triterpenoid scaffold with modifiable positions at C–3 and C–28, has emerged as a valuable lead for antibacterial hybrid development [15]. In 2017, Bębenek et al. [27] applied CuAAC to generate 1,2,3–triazole-linked BA derivatives at C–28; for example, compound **4** (Figure 3) achieved minimum inhibitory concentrations (MIC) of 1.95 µM vs. *Escherichia coli* and 0.95 µM vs. *Klebsiella pneumoniae*, with a bactericidal ratio (MBC:MIC) of ≤4. The introduction of a triazole ring at C–28 is believed to enhance antimicrobial activity by increasing molecular polarity and introducing a heteroaryl donor system, facilitating stronger interactions with bacterial membranes and improving cellular uptake via enhanced lipophilicity/hydrophilicity balance.

Advancing this concept, a 2019 study by Bildziukevich et al. [28] synthesised a series of BA derivatives by coupling their C–28 carboxyl group to cystamine via amide bond formation, yielding hybrids that incorporate disulfide bridges. These redox-active moieties are known to undergo thiol–disulfide exchange reactions, which can disrupt bacterial redox homeostasis and compromise membrane integrity. The resulting compound **5** (Figure 3) exhibited potent antibacterial activity, with MIC and MBC values of 3.125 µM against *Streptococcus* mutans and 25 µM against *Bacillus cereus*, significantly outperforming native BA. The enhanced efficacy is attributed to the dual contribution of the disulfide linkage, which facilitates oxidative stress, and the increased aqueous solubility conferred by the cystamine tail, enabling better cellular uptake and interaction with bacterial targets.

Lombrea et al. [11] investigated the antibacterial properties of several hybrid molecules derived from 2,3–indolo–BA, in which glycine moieties were incorporated to improve biological activity against Gram-positive pathogens. Among the synthesised compounds, compound 6 (Figure 3) exhibited enhanced inhibitory effects, particularly against *Streptococcus pyogenes* and *Staphylococcus aureus*. The parent compound, BA, showed no significant activity under the same conditions, while compound 6 demonstrated the most notable performance with MICs of 13 µg/mL and 26 µg/mL against *S. pyogenes* and *S. aureus*, respectively. Advancing BA’s antibacterial potential, a 2024 study by Amin et al. [29] presented a series of BA–dithiocarbamate (DTC) conjugates, evaluated against both *E. coli* and *S. aureus*. All synthesised derivatives and the parent BA demonstrated moderate antibacterial activity, with MIC values ranging from 64 μg/mL to 128 μg/mL. This emphasises that although these BA-based hybrids do not dramatically surpass the native molecule, they offer structural insights for future potency-enhancing modifications. One of these derivatives is compound **7** (Figure 2).

Grymel et al. [30] investigated the antibacterial potential of betulin derivatives functionalized with cationic triphenylphosphonium (TPP^+^) moieties. By attaching TPP^+^ groups to either the C–3 or C–28 positions of betulin via ester or ether linkages, they generated two series of conjugates (**8a**–**d** and **9a**–**c**, presented in Figure 3). These modifications introduced a permanent positive charge, enhancing the compounds’ ability to interact with negatively charged bacterial membranes. Biological evaluation revealed that all TPP^+^-modified hybrids exhibited significantly improved activity against Gram-positive strains. At a concentration of 200 µM, several analogues effectively inhibited the growth of *S. aureus* (ATCC 25923) and *S. epidermidis* (ATCC 12228), whereas unmodified betulin showed no measurable activity under the same conditions. The enhanced efficacy is attributed to the electrostatic attraction between the cationic phosphonium group and the anionic bacterial envelope, facilitating membrane disruption and compound uptake.

In a similar vein, Shakurova et al. [31] prepared quaternary N–heterocycles of BA methyl ester. One-pot quaternization of 28–O–methyl betulinate gave a pyridinium salt (compound **10** in Figure 3) and reduction in this salt yielded a tetrahydropyridine (compound **11** in Figure 3). These permanently charged BA derivatives proved highly active against *S. aureus*: compound **10** had an MIC of 4 µg/mL and compound **11** had an MIC of 16 µg/mL. These findings confirm that introducing a permanent positive charge (via phosphonium or pyridinium motifs) onto the lupane scaffold greatly enhances Gram-positive antibacterial potency, far surpassing the essentially inactive parent BA under the same conditions.

Another recent strategy has been to append 1,2,3–triazole rings to the lupane framework. Jalmakhanbetova et al. [32] reported a series of betulonic acid–triazole hybrids (betulonic acid is the C–3 ketone of BA) and screened them against common bacterial pathogens. Several of these triazole-bearing derivatives showed pronounced activity; for example, compound **12** (Figure 3) was highly active against *S. aureus*, while compound **13** (Figure 3) achieved a very low MIC of 6.3 µg/mL against *E. coli* (ATCC 25922). In other words, adding a triazole linker converted an otherwise modestly active lupane into a dual-spectrum agent. Together with the earlier CuAAC-derived BA-triazoles (e.g., Bębenek et al. 2017 [27]), these results demonstrate that 1,2,3–triazole appendages at the lupane C–28/C–3 positions can endow hybrids with potent inhibition of both Gram-positive and Gram-negative bacteria.

Researchers have explored a wide range of chemical tweaks to BA in hopes of boosting its antibacterial power, and the results are promising. A summary of representative BA–based hybrids, their tested bacterial strains, SAR, and mode of action is represented in Table 1. Most strategies focus on modifying the C–28 position, where adding triazole rings, redox-active disulfides, or cationic groups like triphenylphosphonium and pyridinium has led to stronger activity, especially against Gram-positive bacteria like *S. aureus*. Some hybrids, like those with triazole or indole–glycine combinations, also show good performance against Gram-negative strains and biofilms. These changes help the molecules interact better with bacterial membranes, increase solubility, and sometimes trigger oxidative stress inside the cells. While not every modification outperforms native BA, the overall trend is clear: smart structural additions at key positions can turn a modest natural compound into a potent antibacterial agent with broader reach and improved uptake.

#### 3.1.3. Antifungal Improvements of Betulinic Acid-Based Hybrid Molecules

**Figure 4:**
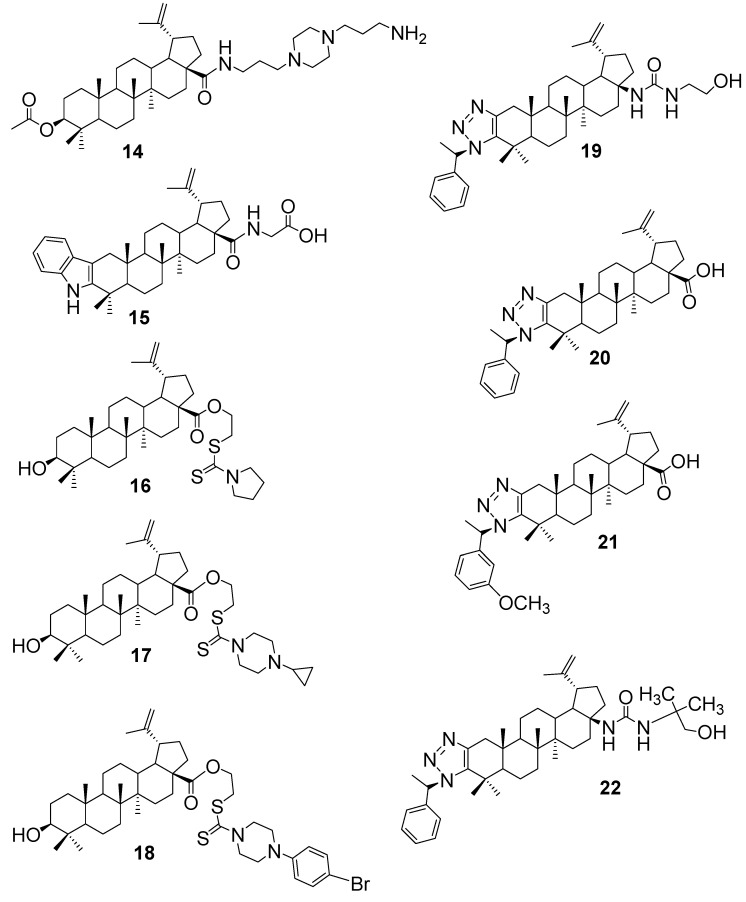
Betulinic acid-based hybrid structures with antifungal activity.

Antifungal agents are designed to inhibit or kill pathogenic fungi, addressing infections ranging from superficial mycoses to life-threatening systemic diseases [33]. In 2019, Krummenauer and colleagues [34] developed a semi-synthetic derivative of BA, referred to as compound **14** (Figure 4), specifically designed to tackle *Cryptococcus neoformans* and *C. gattii*, two fungal pathogens notorious for forming resilient biofilms. By modifying the C–28 carboxyl group with an aromatic ester, they enhanced the compound’s ability to penetrate fungal biofilms and interact with the cell wall. What makes this hybrid particularly promising is its dual performance: it not only disrupted mature biofilms but also maintained low toxicity toward mammalian cells. In antifungal assays, compound **14** showed strong activity, with both MIC and MFC values of 11.4 µM against *C. neoformans* strains H99 and B3501, and 7.6 µM against *C. gattii* R265. These results suggest that the hybrid could be a valuable lead in developing treatments aimed at biofilm-associated fungal infections, especially where conventional drugs fall short.

In 2023, Lombrea et al. [11] introduced a novel class of 2,3–indolo–BA derivatives, aiming to enhance the bioactivity of the lupane scaffold through strategic A–ring fusion and amino acid conjugation. Among the synthesised compounds, the glycine-modified analogue compound **15** (Figure 4) stood out for its antifungal efficacy. This hybrid demonstrated inhibitory activity against *C. albicans* and *C. parapsilosis*, with MIC values of 29 µg/mL for both strains. The incorporation of a glycine moiety at C–28, combined with the indole fusion at C–2/C–3, likely improved the compound’s solubility and membrane interaction, enabling it to overcome the inherent limitations of native BA. Notably, this study was the first to report antifungal activity within this specific scaffold class, highlighting its potential as a foundation for further antifungal drug development.

The most significant improvement came in 2024, when Amin and co-authors [29] developed BA–dithiocarbamate conjugates; the lead compound **16** (Figure 4) achieved an MIC of 4 µg/mL, followed by compounds **17** and **18**, both achieving 32 µg/mL against *C. albicans*. Compound **16** showed synergy with fluconazole and nystatin, inhibited hyphal formation, and completely eradicated fungal cells within 8 h at 8× MIC, underscoring the potency gains achievable through targeted sulfur-based modifications. Notably, its minimum fungicidal concentration equalled its MIC, whereas standard drugs typically required higher doses to kill fungal cells. In summary, the dithiocarbamate conjugates, particularly compound **16**, substantially improved antifungal potency over BA itself, illustrating a successful strategy to augment lupane triterpenes’ activity against fungal pathogens.

Jalmakhanbetova et al. [32] reported a series of BA derivatives bearing 1,2,3–triazole rings, and they observed that specific halogenated analogues showed moderate gains in antifungal efficacy. In particular, a fluorine-containing triazole derivative (compound **13** in Figure 3) was active against *C. albicans* with an MIC of 25 µg/mL, twice as potent as the parent BA (MIC of 50 µg/mL). Similarly, another triazole analogue (compound **19** in Figure 4) displayed an MIC of 25 µg/mL vs *C. albicans* (versus 50 µg/mL for its original precursor). Compounds **20**, **21** and **22** (Figure 4) showed an antifungal effect against *C. albicans* with an MIC of 50 µg/mL, the same as the original precursor. So far, introducing a fluorine atom (and an ethanolamine side chain) into the triazole-modified BA generally doubled antifungal activity compared to non-halogenated versions. Thus, triazole conjugation, especially with electron-withdrawing (halogen) substituents, led to measurable (roughly 2×) improvements in inhibiting *Candida* relative to the unmodified lupane acid.

Recent advances in BA chemistry have led to the development of hybrid molecules with significantly improved antifungal properties. A summary of representative BA–based hybrids, their evaluated fungal strains, SAR, and mode of action is represented in Table 2. Most enhancements stem from modifications at the C–28 position, where attaching aromatic esters, indole–amino acid tails, sulfur-rich dithiocarbamates, or halogenated triazoles have yielded compounds that outperform native BA. Some hybrids, like compound **14** (Figure 4), effectively disrupt fungal biofilms with low toxicity, while others, such as compound **16** (Figure 4), achieve rapid fungicidal action and synergize with standard drugs like fluconazole. Triazole-linked derivatives also show promising gains, especially when halogen atoms are introduced to boost membrane targeting. Overall, these structural upgrades have transformed BA from a modest natural antifungal into a versatile scaffold capable of tackling resistant fungal strains like *C. albicans* and *C. neoformans*.

#### 3.1.4. Structure–Activity Relationships (SARs) of Betulinic Acid Hybrids

BA exhibits tuneable biological activity through structural modifications mainly at the C–28 carboxyl, C–3 hydroxyl, and, to a lesser extent, the ring carbons (C–2, C–20, C–29). Adjustments at these regions influence the molecule’s polarity, ionisation, and ability to interact with microbial membranes or enzymatic targets, thereby determining its antibacterial and antifungal potency [11,31].

C–28 conjugation remains the most reliable handle for potency improvement. Amide, triazole, peptide, and sulfur-rich DTC linkers at this position consistently deliver stronger activity than the parent compound. Among these, BA–DTC hybrids have shown remarkable antifungal effects, achieving *C. albicans* MIC values around 4 µg/mL, inhibition of hyphal development, and synergy with standard antifungals such as fluconazole and nystatin [29]. This enhancement is attributed to the amphiphilic and sulfur donor nature of the DTC moiety, which facilitates membrane disruption and improves drug uptake. Likewise, triazole-linked C–28 derivatives display low-micromolar antibacterial activity across Gram-positive and Gram-negative strains, suggesting that electron-rich heterocycles at this site enhance permeability and target affinity [30]. C–3 and A–ring modifications serve mainly to fine-tune physicochemical balance and selectivity. Indole-fused BA hybrids substituted at C–2/C–3 and bearing glycine or oligo–glycine amide tails at C–28 exhibit clear improvements in activity against *Candida* spp. and Gram-positive bacteria, while native BA remains largely inactive [11]. These dual modifications combine π–surface expansion from the indole nucleus with the ionisation control of the C–28 amide, yielding hybrids that are both more lipophilic and more water-compatible. Other studies show that mild acylation or glycosylation at C–3 improves aqueous solubility and enhances biofilm inhibition without compromising potency [31].

Cationic substitution through amine, guanidine, pyridinium, or phosphonium groups has proven especially effective against encapsulated yeasts and Gram-positive pathogens. Such modifications, typically installed at C–28 or C–3, enable stronger electrostatic attraction to negatively charged microbial surfaces and promote intracellular accumulation [14,31]. Triphenylphosphonium (TPP^+^) and pyridinium conjugates, for instance, exhibit sub-µg/mL MIC values against *S. aureus* and *C. neoformans*, highlighting the benefit of a permanent positive charge on membrane-associated activity [14,30]. Sulfur-containing and redox-active linkers, including cystamine amides and DTC groups, also produce fungicidal or bactericidal effects. These hybrids display MIC and MFC values that coincide, indicating direct membrane disruption rather than metabolic inhibition [29,35]. Their enhanced amphiphilicity and donor characters are believed to contribute to synergy with azole and polyene antifungal drugs. Occasional ring modifications, such as planarizing indole fusions or small heterocyclic insertions, can expand the activity spectrum and enable biofilm eradication in resistant fungal species [35]. However, these structural edits are less predictable and require individual optimisation compared to the more reliable C–28 or cationic strategies.

Overall, studies on BA hybrids reveal a coherent SAR pattern linking structural modification to biological response. The C–28 carboxyl group is the most sensitive and productive site for derivatization, where attachment of amide, triazole, or sulfur-containing linkers markedly enhances antimicrobial potency and often converts a fungistatic scaffold into a fungicidal one. Adjustments at the C–3 position or within the A–ring primarily refine solubility, selectivity, and biofilm penetration rather than drive large potency shifts, though these modifications become powerful when combined with C–28 handles. Introduction of cationic or redox-active fragments further strengthens activity against encapsulated yeasts and Gram-positive bacteria by facilitating electrostatic binding and membrane disruption. Collectively, these findings establish a clear medicinal chemistry roadmap: C–28 functionalization defines potency, C–3 and A–ring tailoring improve pharmacological balance, and cationic motifs ensure broad-spectrum reach, together guiding the rational design of the next generation of BA-based antimicrobial agents.

### 3.2. Oleanolic Acid-Based Hybrid Molecules

#### 3.2.1. Synthetic Approaches to Oleanolic Acid-Based Hybrid Molecules

OA hybrids are most often prepared by derivatizing the 3β–hydroxyl and/or the 28–carboxyl groups on the triterpene scaffold [14]. For example, the C–28 acid is readily esterified or amidated (e.g., via DCC/EDC coupling) to append alcohol or amine fragments [12,36]. Using this strategy, OA has been linked via amide bonds to heterocycles, NSAIDs or other acid fragments to form hybrid esters or amides [12,35]. In parallel, glycosylation (often via “neoglycosylation”) has been applied at either C–3 or C–28 to install sugar moieties; for example, O–methyl glycoside derivatives were obtained by reacting OA with activated sugars at those positions [37]. Another common tactic is CuAAC: for instance, OA’s 3–hydroxyl can be esterified with an alkynoic acid and then cycloadded with aromatic azides to give 1,2,3–triazole-linked hybrids [12]. Similarly, dithiocarbamate conjugation has been achieved via a two-step route in which the C–28 carboxyl is first transformed into an ethylidene bromide and then treated with CS_2_ and a secondary amine to yield OA–dithiocarbamate adducts [38].

Other reported transformations include oxidation of C–3 to a ketone and conversion to oximes, followed by further esterification (“iminoester” hybrids) [39,40], and the formation of OA dimers via amide, ester or triazole linkages [35]. These varied synthetic methods (glycosylations, amide/ester couplings, click cycloadditions, dithiocarbamates, etc.) thus expand the OA framework at the key functional handles (especially C–3 and C–27), enabling a broad array of hybrid structures with improved biological profiles [12,14,35,36,38,39]. More recent approaches introduced polyamine substituents at C–3 and C–17 positions, yielding conjugates capable of disrupting bacterial membranes and enhancing antibiotic efficacy [35]. Beyond the major functional handles, less conventional modifications have been reported, such as Claisen–Schmidt condensation at C–2 to produce arylidene hybrids with improved anti-inflammatory activity [9], highlighting the chemical versatility of the scaffold. Collectively, these synthetic efforts demonstrate that targeted modifications at C–3, C–28, and occasionally C–2 enable the construction of structurally diverse hybrids with improved pharmacological potential over native OA.

OA chemistry centres on exploiting the C–3 hydroxyl and C–28 carboxyl for esterification, amidation, glycosylation or CuAAC reactions. These reactions are modular and reliably generate diverse libraries (e.g., triazoles, dithiocarbamates, polyamine conjugates), which facilitates rapid SAR exploration and incorporation of membrane-active or heteroaromatic pharmacophores. Consequently, OA is an adaptable scaffold for hybrid design when targeted modifications at C–3 or C–28 are prioritised.

#### 3.2.2. Antibacterial Improvements of Oleanolic Acid-Based Hybrid Molecules

**Figure 5:**
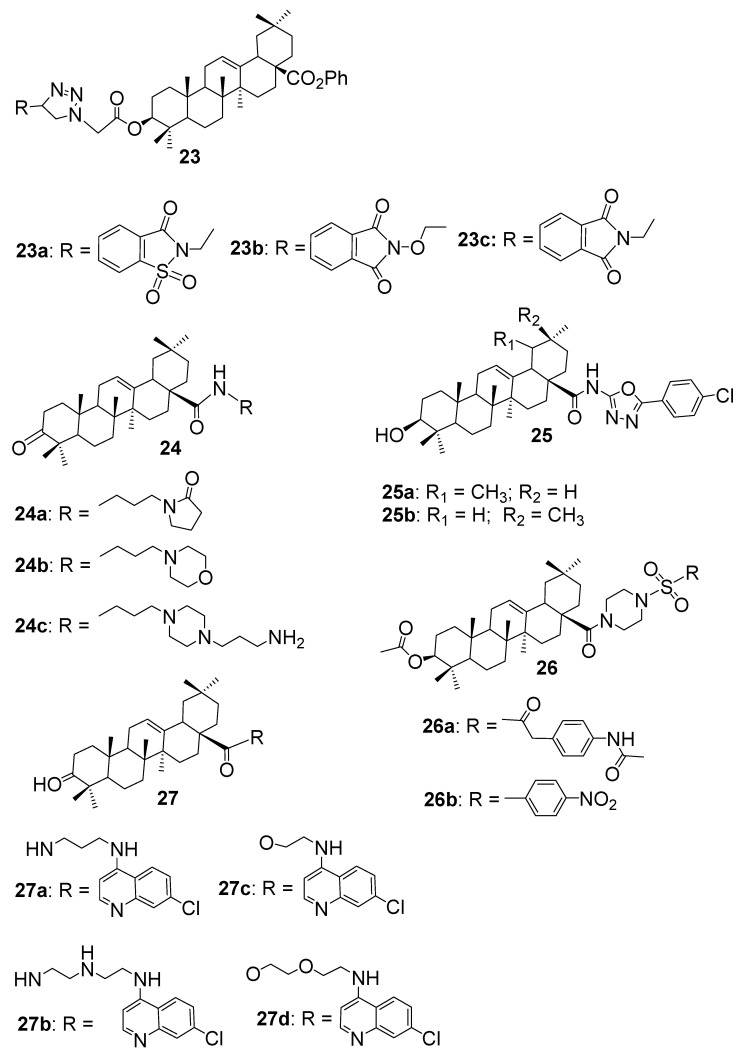
Oleanolic acid hybrids with antibacterial activity.

OA is a well-characterised triterpenoid, which gained renewed interest as a scaffold for antibacterial hybrid design due to its modifiable functional groups and broad-spectrum baseline activity [9,12,35]. In a study, Lahmadi et al. [12] synthesised a series of OA hybrids by tethering phthalimidine and 1,2,3–triazole fragments to the OA backbone, aiming to enhance its antibacterial spectrum. These hybrids were evaluated against four clinically relevant bacterial strains: *S. aureus* ATCC 25923, *L. monocytogenes* ATCC 19115, *S. typhimurium* ATCC 14080, and *P. aeruginosa* ATCC 27853. Interestingly, the parent OA showed moderate inhibition against *S. aureus*, *S. typhimurium*, and *P. aeruginosa*, but was completely inactive against *L. monocytogenes*. Among the newly synthesised compounds, **23a**, **23b**, and **23c** (Figure 5) demonstrated the strongest activity against *L. monocytogenes*, with MIC values of 9.48, 9.56, and 9.89 μM, respectively. Their corresponding MBCs were significantly higher, ranging from 151.86 to 633.66 μM, suggesting a bacteriostatic mode of action. Notably, these MIC values were substantially lower than those of reference antibiotics like tetracycline (576 μM) and chlorhexidine (253 μM), underscoring the potential of OA–triazole hybrids as promising leads for Gram-positive antibacterial development.

Khusnutdinova et al. [35] explored a focused series of OA amide hybrids bearing heterocyclic moieties at the C–28 position to enhance antimicrobial potential. Among these, compounds **24a**, **24b**, and **24c** (Figure 5), featuring pyrrolidinone, morpholine, and piperazine rings, respectively, demonstrated distinct improvements compared with the parent OA scaffold. Notably, **24c**, the piperazine conjugate, exhibited the most promising activity across both Gram-positive and Gram-negative strains, achieving MIC values as low as 12.5 µM against *S. aureus*, *E. faecalis*, and *B. cereus*, and maintaining moderate efficacy (25–50 µM) against *E. coli*, *S. enterica*, and *P. aeruginosa*. This outcome highlights the beneficial role of the piperazine ring in promoting optimal lipophilic–hydrophilic balance and facilitating bacterial membrane interaction. In contrast, **24a** and **24b** analogues exhibited no measurable activity, with all reported MICs > 200 µM, although several strains not tested for **24a** at least showed an activity of ≤200 µM against *S. faecalis*. Several of the non-heterocyclic polyamine conjugates in the same series also exhibited enhanced antibacterial profiles, suggesting that both chain length and amine functionality play critical roles in optimising the bioactivity of OA-derived hybrids.

Khwaza et al. [41] investigated a broader set of antimicrobial hybrids, several of which incorporated triterpenoid cores such as OA. Among these, compounds **25a** and **25b** (Figure 5) featured OA conjugated to distinct bioactive fragments. Compound **25a**, an OA amide-linked to a chlorophenyl–oxadiazole moiety, showed relatively weak antibacterial performance, with MIC values ranging from 125 to 250 µg/mL against Gram-positive strains and only moderate inhibition of Gram-negative bacteria. In contrast, compound **25b** demonstrated improved activity against several Gram-negative pathogens, including *E. cloacae*, *P. vulgaris*, *K. oxytoca*, *P. aeruginosa*, and *P. mirabilis*, with MIC values between 15.6 and 31.2 µg/mL. Notably, its MIC against *K. pneumoniae* was 62.5 µg/mL, better than its activity against *E. coli*. Although MBC values were not reported, the study emphasised that ciprofloxacin-linked hybrids (MIC = 7.8 µg/mL) and phenolic–monoterpenoid conjugates were the most potent overall. Compared to these, the OA-based compound **25a** was confirmed to be only moderately active, underscoring the importance of pharmacophore selection in hybrid design.

Ma et al. [42] designed a new class of OA 28–piperazine sulfonamide derivatives and evaluated their in vitro antibacterial activity against three phytopathogens, including *Xanthomonas oryzae pv. oryzicola* (XOO), *Xanthomonas axonopodis pv. citri* (XAC), and *Pseudomonas syringae pv. actinidiae* (PSA). Among the synthesised analogues, the piperazine-linked compounds **26a** and **26b** (Figure 5) stood out for their potent inhibitory effects, particularly against XAC. At 200 µg/mL, both displayed inhibition rates exceeding 83%, surpassing the reference thiodiazole–copper (82%), and retained high activity even at 100 µg/mL. Their half-maximal effective concentrations (EC_50_) further confirmed this potency, measuring 31.4 µg/mL for **26a** and 45.1 µg/mL for **26b**, both lower than the control (56.4 µg/mL). Comparable performance was observed against XOO and PSA, highlighting their broad-spectrum efficacy. The incorporation of the piperazine–sulfonamide fragment appears to enhance the amphiphilicity and electronic character of the OA scaffold, leading to improved bacterial membrane interactions and overall antimicrobial performance.

Khwaza et al. [13] synthesised a series of OA–4–aminoquinoline hybrid compounds to enhance antibacterial efficacy against both Gram-positive and Gram-negative strains. Among these, compounds **27a**, **27b**, and **27c** (Figure 5) displayed notable broad-spectrum activity, particularly against *E. faecalis*, *Klebsiella oxytoca*, and *E. coli*, with MIC values of 1.25 mg/mL, approximately two times more potent than the parent OA scaffold (MIC = 2.5 mg/mL). Compounds **27b** and **27c** further demonstrated enhanced inhibition of *S. aureus*, while **27a** and **27b** effectively suppressed *E. cloacae* and *P. vulgaris* at the same concentration. Compound **27d** (Figure 5) showed a more selective profile, exhibiting strong activity against *E. faecalis* and *E. coli* (MIC = 1.25 mg/mL) while maintaining moderate effects on other tested strains. These results highlight that conjugation with the 4–aminoquinoline fragment can significantly improve the antibacterial properties of the OA scaffold, and that subtle variations in the hybrid structure influence both potency and spectrum of activity.

This summarises how chemical modifications to OA have led to meaningful gains in antibacterial activity. A summary of representative OA–based hybrids, their evaluated bacterial strains, SAR, and mode of action is represented in Table 3. Most improvements come from tweaking the C–3 hydroxyl and C–28 carboxyl groups, allowing researchers to attach pharmacophores like triazoles, dithiocarbamates, phthalimidine–triazole linkers, and even antibiotic fragments. These hybrids have shown enhanced potency against both Gram-positive and Gram-negative bacteria. For instance, compounds **23a**–**c** outperformed standard antibiotics like tetracycline against *Listeria monocytogenes*, while compound **25b** showed selective activity against *Klebsiella pneumoniae* and *Pseudomonas aeruginosa*. Although some hybrids, like **25a**, were only moderately active, the overall trend confirms that strategic structural additions, especially those that improve membrane interaction or introduce redox-active groups, can significantly boost OA’s antibacterial potential.

#### 3.2.3. Antifungal Improvements of Oleanolic Acid-Based Hybrid Molecules

**Figure 6:**
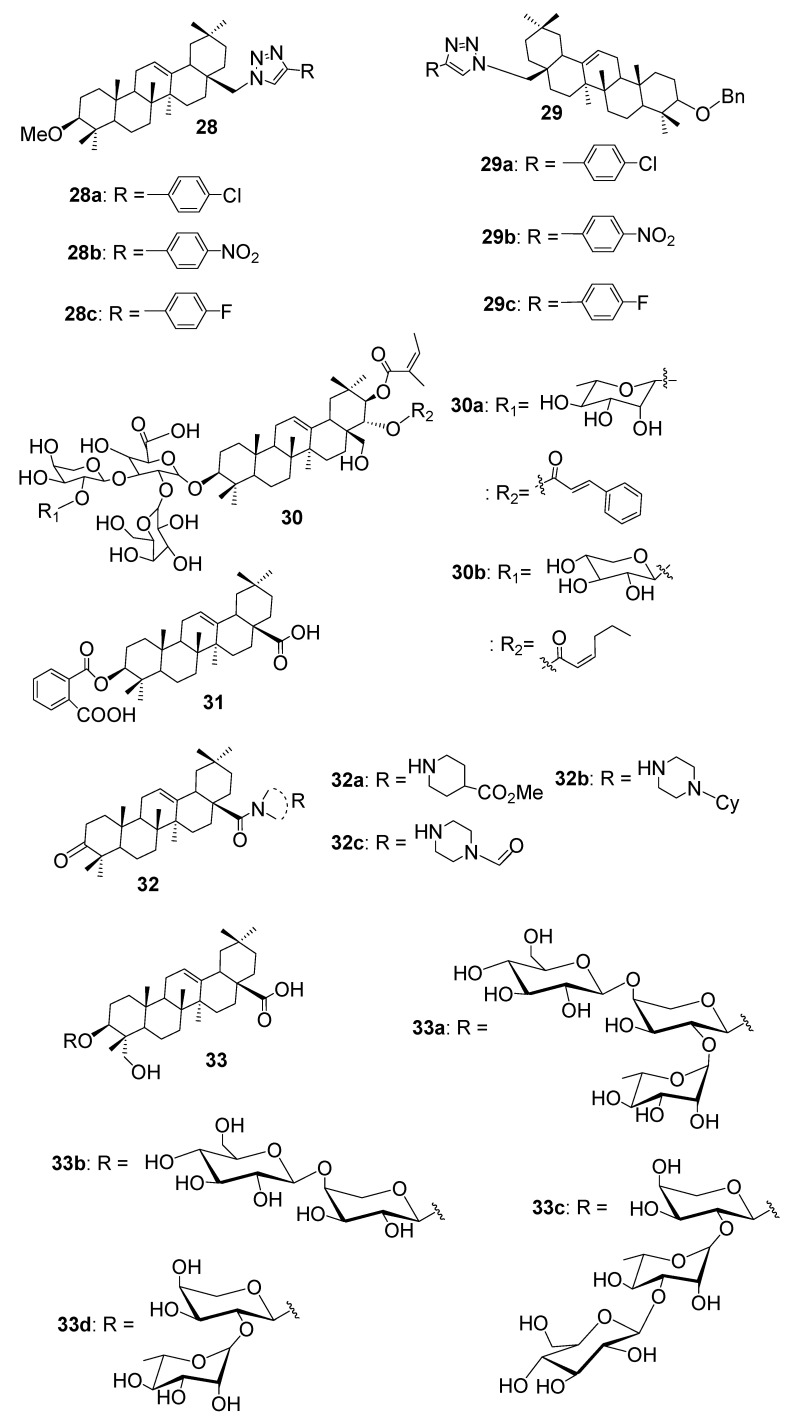
Oleanolic acid hybrids with antifungal activity.

Several semisynthetic oleanane derivatives have demonstrated notable antifungal improvements over native OA. In a research conducted by Chen et al. [43], evaluated a series of oleanane-type triterpene–1,2,3–triazole conjugates for antifungal activity against six phytopathogenic fungi. Among the series, compounds **28a**, **28b**, **28c**, **29a**, **29b**, and **29c** (Figure 6) exhibited the highest inhibitory effects, particularly against *Sclerotinia sclerotiorum*, achieving inhibition rates of 85.6%, 83.1%, 87.6%, 86.8%, 87.7%, and 89.6%, respectively, at 50 µg/mL. These compounds also showed substantial activity against *Botrytis cinerea* and *Rhizoctonia solani*, with inhibition generally exceeding 70%, and moderate effects against the remaining fungi. The parent OA displayed much lower activity, with inhibition rates below 21% across all species. Structure–activity analysis indicated that the presence of electron-withdrawing substituents on the phenyl ring (Cl, NO_2_, F) enhanced antifungal potency, and that compounds bearing a benzyl group (compound **29**) were generally more active than their methyl-substituted counterparts (compound **28**). These results demonstrate that conjugation of triazole fragments and careful tuning of aromatic substituents can markedly improve the fungicidal properties of OA derivatives.

Most recently, Zong et al. [44] reported the isolation of two novel oleanane-type triterpenoid saponins compounds **30a** and **30b** (Figure 6) from the flowers of *Camellia sinensis*. These saponins were structurally characterised by glycosidic linkages at C–3 and hydroxyl substitutions that enhance aqueous solubility and membrane interaction. When tested against *C. albicans*, compound **30a** exhibited an MIC of 5.06 µM and compound **30b** 7.81 µM, both closely matching the potency of fluconazole (MIC = 4.25 µM). Remarkably, all four saponins evaluated in the study including two previously known analogues showed stronger inhibitory activity against *C. glabrata* than fluconazole, highlighting their potential as leads for treating drug-resistant fungal infections. Enhanced efficacy is likely due to the amphiphilic nature of the saponin framework, which facilitates membrane disruption and ergosterol interference.

Among the OA derivatives synthesised by Wei et al. [45], compound **31** (Figure 6) displayed a remarkable improvement in antifungal potency compared with the parent molecule. The incorporation of a terminal carboxyl group enhanced its hydrophilic balance, which likely strengthened interactions with fungal cell membranes. Compound **31** exhibited a MIC of 8 µg/mL against *C. albicans*, a substantial enhancement over OA (MIC > 64 µg/mL). This result highlights that strategic hydrophilic modification of the oleanane scaffold can significantly enhance antifungal performance and broaden the potential of OA as a platform for antifungal drug design.

Sui et al. [46] synthesised a range of OA-based tertiary amide derivatives to enhance antifungal potency against key phytopathogenic fungi, including *Fusarium graminearum*, *Gaeumannomyces graminis*, *Colletotrichum orbiculare*, and *Valsa mali*, evaluated at 50 µg/mL. Among these, compounds **32a**, **32b**, and **32c** (Figure 6) exhibited remarkable improvements in antifungal activity compared to the parent OA. Compound **32a** demonstrated a significant inhibitory effect against *G. graminis* with an inhibition rate of 50.0%, indicating a substantial enhancement relative to the weak performance of unmodified OA. Similarly, compound **32b** showed a potent inhibition of *G. graminis* at 52.4%, underscoring its effectiveness in suppressing fungal mycelial growth. The most promising derivative, **32c**, displayed strong and broad-spectrum antifungal activity, achieving inhibition rates of 53.2% against *G. graminis* and 63.0% against *V. mali*. Further quantitative evaluation confirmed that **32c** had EC_50_ values of 41.77 µg/mL and 43.96 µg/mL against *G. graminis* and *V. mali*, respectively, which, although higher than carbendazim (EC_50_ = 2.67 and 0.93 µg/mL), represent a meaningful advancement for OA derivatives. These results clearly indicate that introducing tertiary amide functionalities into the OA scaffold significantly enhances its antifungal potency, particularly against *G. graminis* and *V. mali*, positioning compounds such as **32a**, **32b**, and especially **32c** as promising leads for further development of antifungal agents targeting phytopathogenic fungi.

In a 2024 study by Tan et al. [47], the antifungal potential of OA-type saponins isolated from *Pulsatilla chinensis* was evaluated against three pathogenic fungi: *C. albicans*, *C. neoformans*, and *C. parapsilosis*. Among the saponins tested, compounds **33a**, **33b**, **33c**, and **33d** (Figure 6) demonstrated notable antifungal activity. Compound **33a** exhibited MICs of 12.5 μg/mL against *C. albicans* and 25 μg/mL against both *C. neoformans* and *C. parapsilosis*. Similarly, compound **33b** showed MICs of 12.5 μg/mL for *C. albicans* and 25 μg/mL for the latter two fungi. Compounds **33c** and **33d** mirrored these results, with MICs of 12.5 μg/mL against *C. albicans* and 25 μg/mL against *C. neoformans* and *C. parapsilosis*, highlighting their consistent and potent antifungal effects. These findings indicate that the presence of a disaccharide or trisaccharide moiety at C–3 and a free hydroxyl group at C–23 are critical structural features contributing to the enhanced antifungal activity of these OA-type saponins. Compared to other saponins with modifications at these positions or aglycone forms, these compounds displayed superior activity, underscoring the importance of glycosylation and hydroxylation in optimising antifungal efficacy.

This section highlights how modifying OA has led to promising antifungal hybrids, especially against *Candida* species. A summary of representative OA–based hybrids, their evaluated fungal strains, SAR, and mode of action is represented in Table 4. Researchers have explored various structural additions, such as phthalimidine–triazole linkers, oxadiazole fragments, and saponin-like glycosides to improve OA’s potency and spectrum. Some hybrids, like compounds **30a** and **30b**, matched or even surpassed fluconazole in activity against *C. albicans* and *C. glabrata*. Others, like compound **15** (Figure 4) from Lombrea’s work, introduced amino acid tails and indole fusions to boost membrane interaction and solubility. While not all OA derivatives outperformed standard antifungals, many showed low cytotoxicity and strong biofilm inhibition, suggesting that OA remains a valuable scaffold for designing next-generation antifungal agents.

#### 3.2.4. Structure–Activity Relationships of Oleanolic Acid Hybrids

The structure–activity data reported for OA hybrids reveal a clear relationship between specific chemical modifications and improvements in antimicrobial performance. Most derivatives achieve enhanced potency through substitutions at the C–28 carboxyl and C–3 hydroxyl groups, which alter the compound’s amphiphilic balance, charge distribution, and capacity to interact with microbial membranes or intracellular targets [39,43,46].

The C–28 position remains the most responsive site for derivatization, as the introduction of amide, triazole, piperazine, or sulfur-containing linkers consistently yields higher antibacterial and antifungal activity compared to the parent scaffold. For instance, phthalimidine-tethered triazole hybrids displayed MIC values around 9.5 μM against *L. monocytogenes*, while piperazine-bearing amide derivatives achieved MICs near 12.5 μM against *S. aureus*, *E. faecalis* and *B. cereus* [39,43]. These results suggest that moderately basic or heteroaromatic moieties at C–28 can strengthen electrostatic and hydrophobic interactions with bacterial membranes, producing faster or more sustained inhibitory effects. Similar observations were reported for oxadiazole and chlorophenyl conjugates, where electron-withdrawing substituents further improved antibacterial efficiency by facilitating membrane permeation and potential interaction with protein targets [44]. Adjustments at the C–3 position, including mild esterification and glycosylation, generally fine-tune solubility, selectivity, and biofilm inhibition rather than generate major potency shifts. Glycosylated OA derivatives, particularly natural and semisynthetic saponins, exhibit strong antifungal properties that rival or exceed fluconazole. Compounds such as camellia saponins produced MIC values of 5.06 and 7.81 μM against *C. albicans* and *C. glabrata*, respectively, demonstrating that the attached sugar units enhance water compatibility and promote membrane-active fungicidal effects [47]. Wei et al. reported that incorporation of an additional terminal carboxyl increased hydrophilicity and led to an eight-fold improvement in antifungal potency relative to native OA) [45].

Derivatives carrying cationic or polyamine functionalities, such as amine, guanidine, pyridinium, or phosphonium fragments, showed significant improvement against Gram-positive and encapsulated fungal species. These moieties, usually attached through C–28 amidation, strengthen electrostatic attraction to negatively charged microbial surfaces and facilitate cell entry, leading to MICs in the low-micromolar range [39,45]. In several series, the combination of lipophilic triterpenoid cores with cationic or heteroaromatic substituents produced broad-spectrum hybrids capable of acting on both membranes and intracellular targets. Triazole-based conjugates bearing electron-withdrawing groups such as chlorine, nitro, or fluorine exhibited over 80% mycelial inhibition at 50 µg/mL against *S. sclerotiorum*, illustrating the contribution of aryl-ring electronics to fungicidal potency [48].

Although the C–28 and C–3 sites provide the most predictable improvements, structural diversification of the OA core occasionally produces exceptional outcomes. Compounds with planar or extended aromatic fragments have shown activity against resistant or phytopathogenic fungi, but these effects are highly context–dependent and require individual optimisation [48,49]. Excessive hydrophobicity or bulky substituents can reduce aqueous solubility and limit bioavailability, underscoring the importance of maintaining physicochemical balance when designing new hybrids. Taken together, the collective evidence from recent studies indicates that C–28 functionalisation defines the primary potency axis, while C–3 and A–ring modifications refine solubility and selectivity. Incorporating basic or electron-poor heterocycles further expands the antimicrobial spectrum by enhancing membrane and target engagement. These consistent SAR patterns establish a rational framework for future design: begin with modular C–28 derivatisation to secure core potency, then fine-tune C–3 substitution and heterocycle electronics to optimise activity, selectivity and pharmacological behaviour across bacterial and fungal models [39,43,45,46,48,49,50].

Overall, the structure–activity trends of OA hybrids demonstrate that targeted modification at the C–28 carboxyl position is the key determinant of antimicrobial potency, while substitution at the C–3 hydroxyl or A–ring provides complementary control over solubility, selectivity, and pharmacokinetic balance. Hybrids bearing electron-withdrawing or cationic heterocycles benefit from stronger electrostatic and hydrophobic interactions, translating into broader and more potent antibacterial and antifungal profiles. Collectively, these observations confirm that combining rational C–28 derivatization with strategic tuning of C–3 polarity and side-chain electronics offers a reliable route to designing next-generation oleanane-based antimicrobial agents with improved spectrum and efficacy.

### 3.3. Ursolic Acid-Based Hybrid Molecules

#### 3.3.1. Synthetic Approaches to Ursolic Acid-Based Hybrid Molecules

Ursolic acid (UA, 3β–hydroxy–urs–12–en–28–oic acid) is a pentacyclic triterpenoid commonly sourced from apple peels and various medicinal plants such as rosemary (*Rosmarinus officinalis, syn. Salvia rosmarinus*) and sage (*Salvia officinalis*) [49,50], but its low natural abundance often necessitates semisynthetic access or isolation from agricultural residues [5,6]. Like other triterpenic acids, UA contains two principal functional groups that serve as versatile handles for derivatization: the C–3 hydroxyl and the C–28 carboxyl [48]. The C–28 acid is frequently used for amide or ester formation with amines, amino acids, peptides, or heterocycles under standard coupling conditions (e.g., EDC, DCC), generating hybrids with improved polarity and conjugation opportunities [51]. Another common modification involves 1,2,3–triazole formation via CuAAC, where the C–28 is first converted to an alkyne derivative and subsequently cycloadded with aromatic azides to afford stable triazole-linked hybrids [6].

In parallel, the C–3 hydroxyl has been exploited for esterification or etherification, often using acid chlorides, anhydrides, or activated sugars to furnish glycosides and saponins [52]. Such glycosylation can enhance aqueous solubility and bioavailability, while retaining or modulating antimicrobial potential [53]. Less conventional strategies include oxidation of C–3 to a ketone followed by oximation or imine/oxime ester formation, providing alternative scaffolds for further functionalization [48]. More recently, dithiocarbamate conjugation at C–28 has been developed through a two-step protocol, involving activation of the carboxyl followed by CS_2_/amine treatment, producing sulfur-rich hybrids with expanded pharmacological scope [10]. Beyond the canonical C–3 and C–28 handles, occasional modifications at C–2 and C–17 have been reported, including Claisen–Schmidt condensations and amide couplings, which introduce extended aromatic or polyamine substituents and further diversify the ursane framework [54]. Collectively, these approaches highlight that UA, like its lupane and oleanane counterparts, is most amenable to modification at C–3 and C–28, with emerging chemistries at less common positions offering additional opportunities for hybrid design [6,23].

UA is handled similarly to OA and BA with C–3 and C–28 as the principal derivatization points. Amide/ester formation, CuAAC triazole formation, and glycosylation are the most commonly exploited transformations; emerging strategies (deep ring edits) expand the chemical space but require more extensive validation. Overall, UA offers flexible chemistry for generating hybrids tailored to either potency (C–28 cationic motifs) or pharmacokinetic/solubility improvements (C–3 glycosylation).

#### 3.3.2. Antibacterial Improvements of Ursolic Acid-Based Hybrid Molecules

UA is a pentacyclic triterpenoid with a well-established antibacterial profile and has served as a versatile scaffold for hybrid design, though its activity is highly sensitive to structural modifications at key positions such as C–3 and C–28 [5,6,10].

**Figure 7:**
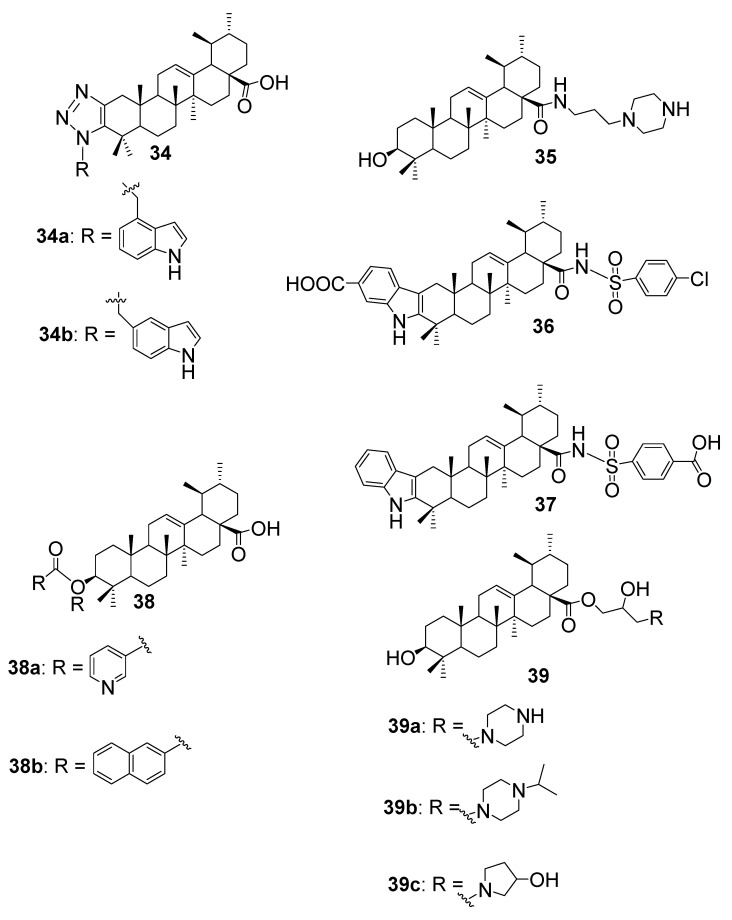
Ursolic acid hybrids with antibacterial activity.

Wang et al. [55] synthesised a series of 1,2,3–triazole-fused UA analogues to explore how heterocyclic fusion affects antimicrobial potency. Among these, compounds **34a** and **34b** (Figure 7) were evaluated against several bacterial strains. Surprisingly, both hybrids showed reduced activity compared to native UA. Specifically, compound **34a** exhibited an MIC of 1.56 mg/mL against *S. aureus*, while **34b** showed an MIC of 27.04 mg/mL against *L. innocua*. Neither compound demonstrated measurable inhibition against *E. coli* or *S. enterica* subsp., mirroring the inactivity of UA itself against these Gram-negative strains. These findings suggest that fusing a triazole ring to the UA scaffold, despite its widespread use in medicinal chemistry, may compromise antibacterial efficacy, possibly due to steric hindrance or altered lipophilicity that impairs membrane interaction and target binding.

Yang et al. [56] synthesised a series of UA derivatives by introducing amide linkages at the C–28 position, aiming to enhance antibacterial potency against phytopathogenic bacteria. Among the tested compounds, the most active analogue is compound **35** (Figure 7), which demonstrated strong inhibitory effects against *X. oryzae* and *X. axonopodis*, with EC_50_ values of 4.42 mg/L and 4.53 mg/L, respectively. These values reflect a substantial improvement over native UA. Mechanistic studies revealed that the amide derivatives triggered intracellular reactive oxygen species (ROS) accumulation, suppressed bacterial antioxidant enzyme activity, and compromised membrane integrity. This combination of oxidative stress and membrane disruption points to a bactericidal mechanism of action, making these UA–amide hybrids promising candidates for the development of eco–friendly agrochemicals targeting bacterial plant pathogens.

In a recent study, Sun et al. [57] synthesised a series of UA derivatives by incorporating 4–chlorobenzenesulfonamide and indole moieties at strategic positions to enhance antibacterial potency. Among these, compounds **36** and **37** (Figure 7) demonstrated exceptional activity against *S. aureus*, including methicillin-resistant strains (MRSA). These hybrids achieved MIC values of approximately 1 µM (≈0.4 µg/mL), significantly outperforming native UA. The structural integration of sulfonamide and indole fragments likely contributed to improved membrane permeability and target binding. Importantly, the compounds exhibited low cytotoxicity and showed strong therapeutic efficacy in mouse models of MRSA infection, suggesting their potential as lead candidates for next-generation anti-MRSA agents.

In the study by Pereira et al. [58], UA ester derivatives **38a** and **38b** (Figure 7) demonstrated significant antibacterial improvements at 100 μg/mL. Compound **38a** exhibited potent inhibition of B. cereus (97.8%), *S. aureus* (53.0%), *E. coli* (87.3%), and *S. typhimurium* (96.3%), indicating a marked enhancement relative to the parent UA. Compound **38b** also showed moderate antibacterial activity, with 51.3% inhibition against *E. coli* and 63.8% against *S. typhimurium*, demonstrating selective improvement against Gram-negative bacteria. These results highlight the effectiveness of esterification in enhancing the antibacterial potential of UA derivatives, with compound **38a** emerging as a particularly promising candidate for broad-spectrum antibacterial applications.

Based on the reported data in Yang et al. [59], the UA derivatives **39a**, **39b**, and **39c** (Figure 7) exhibited notable antibacterial improvements against the plant pathogens *X. oryzae pv. oryzae* (Xoo) and *X. axonopodis pv. citri* (Xac). Compound **39a** showed strong activity with EC_50_ values of 4.77 μg/mL against Xoo and 1.55 μg/mL against Xac, markedly outperforming the parent UA (>100 μg/mL for both pathogens). Derivative **39b**, with increased hydrophobicity at the piperazine tail, demonstrated reduced but still considerable antibacterial potency, with EC_50_ values of 10.60 μg/mL against Xoo and 5.05 μg/mL against Xac. Notably, **39c**, a pyrrolidine-substituted derivative, achieved superior activity, registering EC_50_ values of 4.45 μg/mL against Xoo and 1.39 μg/mL against Xac, highlighting the enhanced bacterial inhibition conferred by the smaller cyclic amine. These results underscore the significant influence of the N-containing side-chain modifications on antibacterial performance, where shorter, less hydrophobic substituents contribute to increased potency. The data clearly indicate that structural tailoring of UA can dramatically improve its efficacy against Gram-negative plant pathogens.

This section outlines how structural modifications to UA have led to notable improvements in antibacterial activity. Researchers have explored various hybrid designs, including 1,2,3–triazole fusions, C–28 amide derivatives, and sulfonamide–indole conjugates. A summary of representative UA–based hybrids, their tested bacterial strains, SAR, and mode of action is represented in Table 5. While some hybrids, like compounds **34a** and **34b**, showed reduced activity against Gram-negative bacteria, others demonstrated significant gains. For instance, compound **35** exhibited strong activity against plant pathogens *X. oryzae* and *X. axonopodis*, with evidence of a bactericidal mechanism involving oxidative stress and membrane disruption. Most notably, compounds **36** and **37**, bearing benzenesulfonamide and indole moieties, achieved low micromolar MICs against *S. aureus*, including MRSA strains, and showed strong in vivo efficacy with minimal toxicity. These findings suggest that while not all UA modifications enhance activity, certain structural motifs, especially those that improve membrane interaction or induce oxidative stress, can significantly boost UA’s antibacterial potential.

#### 3.3.3. Antifungal Improvements of Ursolic Acid-Based Hybrid Molecules

**Figure 8:**
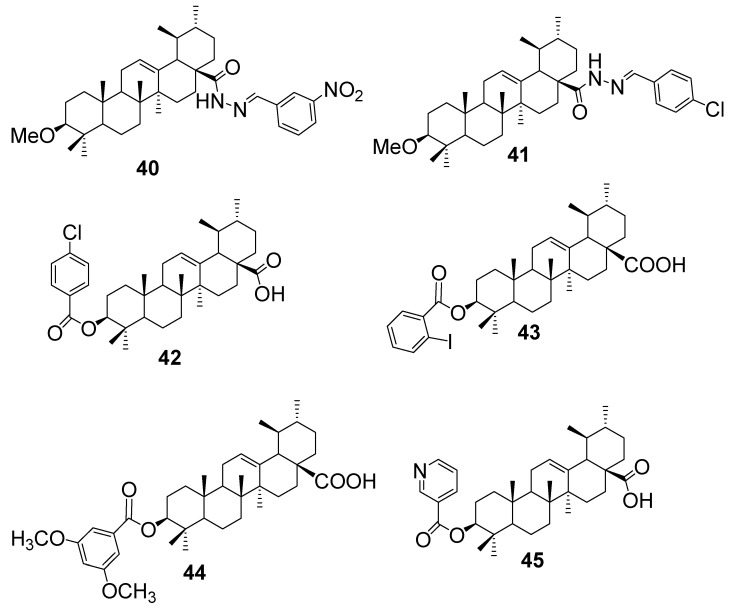
Ursolic acid hybrids with antifungal activity.

UA has long demonstrated modest antifungal activity, but recent hybridization strategies, particularly at the C–3 and C–28 positions, have significantly expanded its potential against resistant fungal strains such as *C. albicans* and *C. neoformans* [55,58]. Şenol et al. [60] synthesised a series of thirteen UA-based arylidene–hydrazide hybrids by modifying the scaffold to include hydrazone linkages at the C–3 position. These derivatives were evaluated for antifungal activity against *C. albicans*. Among them, compounds **40** and **41** (Figure 8) emerged as the most potent, each exhibiting MIC values of 125 mM (approximately 55.7 µg/mL). This represents a notable improvement over native UA, which showed minimal inhibition at comparable concentrations. Although minimum fungicidal concentrations (MFCs) were not reported, the hybrids demonstrated consistent growth suppression at lower doses. The study also benchmarked their activity against amphotericin B, a gold-standard antifungal, but none of the UA-derived hybrids surpassed its potency. Nonetheless, the results underscore the value of arylidene–hydrazide conjugation in enhancing UA’s antifungal profile.

Zhu et al. [61] synthesised and evaluated a series of UA ester derivatives for antifungal and anti-oomycete activity. Among these, compound **42** (Figure 8) demonstrated the most notable improvement in antifungal potency compared with the parent UA. At a concentration of 100 μg/mL, compound **42** inhibited the growth of *P. capsici* and *F. graminearum* by 82.3% and 76.4%, respectively. Both were markedly higher than the inhibition achieved by UA, which showed only 41.6% and 38.7% against the same pathogens. Further quantitative assays revealed that compound **42** exhibited EC_50_ values of 70.5 mg/L against *P. capsici* and 113.2 mg/L against *F. graminearum*, indicating a roughly two-fold increase in activity over the unmodified triterpenoid. Zhu and colleagues [61] attributed this enhanced bioactivity to the esterification at the C–3 hydroxyl position, which improved the compound’s lipophilicity and facilitated stronger interactions with fungal cell membranes. The findings highlight compound **42** as a promising antifungal candidate within the UA framework, demonstrating that strategic structural modifications can substantially enhance antifungal efficacy.

In the work of Pereira et al. [58], a series of UA ester derivatives was synthesised to probe how subtle structural modifications influence antifungal potency. Among the derivatives tested, compounds **43**, **43**, and **45** (Figure 8) demonstrated strikingly enhanced inhibition of *C. albicans*, with respective inhibition rates of 81.1%, 93.1%, and 95.9% at 100 µg/mL. These results reflect a clear structure–activity relationship, where esterification at the C–3 hydroxyl group of the UA skeleton likely increases lipophilicity, thereby improving membrane permeability and facilitating stronger interactions with fungal cell membranes. This enhanced hydrophobic character may disrupt ergosterol-containing lipid bilayers or promote accumulation within the fungal cytoplasmic membrane, compromising cell integrity. Notably, compound **45**, the most active derivative, suggests that specific alkyl or aryl substituents at the ester moiety optimise the balance between molecular rigidity and hydrophobic surface area, maximising antifungal efficacy. Overall, these findings illustrate how strategic chemical derivatization of triterpenoid scaffolds can yield compounds with significantly improved biological activity compared to the parent ursolic acid.

This section summarises the limited but insightful research on enhancing the antifungal properties of UA through hybrid-molecule design. A summary of representative UA–based hybrids, their evaluated fungal strains, SAR, and mode of action is represented in Table 6. Compared to the extensive work on BA and OA derivatives, UA-based antifungal hybrids remain underexplored, with only a few published studies reporting biological evaluations. Among the known examples, arylidene–hydrazide hybrids of UA have shown moderate activity *against C. albicans*, achieving MIC values around 125 mM, markedly better than native UA, though still far below the potency of standard antifungals like amphotericin B. These hybrids were able to inhibit fungal growth at lower concentrations, suggesting some promise despite the absence of minimum fungicidal concentration data. The limited scope of current reports highlights a clear gap in the literature and underscores the need for more systematic exploration of UA’s antifungal potential. From a medicinal chemistry perspective, future efforts could focus on introducing membrane-disruptive motifs, redox-active linkers, or synergistic pharmacophores to improve potency and broaden the spectrum of activity. Overall, while UA offers a chemically versatile scaffold, its antifungal optimisation remains an open frontier requiring deeper structural innovation and mechanistic investigation.

#### 3.3.4. Structure–Activity Relationships of Ursolic Acid Hybrids

Structure–activity data for UA hybrids reveal a pattern broadly comparable to that of OA, yet complexed by the distinct orientation of the C–19 methyl and slightly altered A–ring topology that influences steric accessibility and hydrogen-bonding potential. Across antibacterial and antifungal investigations, modifications at the C–28 carboxyl and C–3 hydroxyl groups dominate the SAR landscape, determining how efficiently the scaffold interacts with microbial membranes and key enzymatic systems [51,52,53,54].

C–28 functionalisation remains the most productive strategy for increasing antimicrobial potency. Amide, triazole, and sulfonamide linkages at this site frequently transform weakly active UA into derivatives with low-micromolar MIC values. Wang et al. [55]. reported UA–triazole hybrids bearing aryl substituents that achieved MICs of 6.25–12.5 µM against *S. aureus* and *E. faecalis*, representing a more than ten-fold enhancement over the parent compound. Yang and co-workers [59] demonstrated that the introduction of short alkyl–amine or piperidine moieties at C–28 produced selective inhibition of Gram-positive bacteria, suggesting that moderate cationic charge combined with increased amphiphilicity enhances adsorption to bacterial cell walls. In addition, C–28 sulfonamide and hydrazide derivatives exhibited dual antibacterial and antifungal properties, underscoring the versatility of this reactive site for hybrid design [60]. Alteration of the C–3 hydroxyl region, although less potent on its own, provides essential control over solubility and membrane interaction. Esterification with small polar fragments or coupling to heteroaromatic residues tends to improve antifungal outcomes by balancing lipophilicity and polarity. Glycosylated UA analogues, for example, display enhanced dispersion in aqueous media and potent antifungal action comparable to fluconazole, likely due to the combined effects of improved permeability and hydrogen-bonding interactions within the fungal cell envelope [48,50].

Cationic and heteroatom-rich appendages again play a decisive role in boosting activity against Gram-positive and encapsulated species. Guanidine- and quaternary-amine-bearing UA conjugates outperform their neutral analogues, achieving sub-10 µM MICs against *B. cereus* and *C. albicans* through stronger electrostatic binding and membrane depolarisation [56,57]. Triazole-linked aromatic fragments substituted with electron-withdrawing groups further enhance fungicidal performance; hybrids carrying chloro- and nitro-phenyl rings exhibit mycelial inhibition exceeding 80% at 50 µg/mL in several phytopathogenic models [58].

While big structural changes to the pentacyclic core, such as ring contractions or additional fused heterocycles, can sometimes unlock novel bioactivity, these transformations often lead to unpredictable outcomes and are less generalisable than targeted C–28 or C–3 derivatisations. Maintaining an optimal balance between the hydrophobic surface area and polar functionality remains critical, as overly bulky or lipophilic side chains tend to reduce solubility and limit cellular availability [55,58,59,60,61]. Taken together, the collective findings indicate that C–28 derivatisation governs potency, C–3 substitution refines physicochemical behaviour, and cationic or electron-withdrawing heterocycles expand the antimicrobial spectrum. Rational design of future UA-based hybrids should therefore prioritise modular C–28 amide or triazole construction, supported by C–3 or glycosidic modifications to improve pharmacological balance, yielding oleanane-type antimicrobials with enhanced efficacy and selectivity across bacterial and fungal pathogens [31,47,57,58,60].

Overall, the SAR patterns of UA hybrids confirm that C–28 derivatization is the principal driver of antimicrobial potency, while C–3 modification plays a supporting role in refining polarity, solubility, and selectivity. The addition of cationic or electron-withdrawing heteroaromatic fragments further enhances interactions with microbial membranes, resulting in broader and stronger antibacterial and antifungal profiles. When these elements are balanced, C–28 amide or triazole linkages for potency, C–3 or glycosidic alterations for pharmacological tuning, and cationic substitutions for cell surface engagement, the resulting hybrids consistently outperform the parent scaffold. Collectively, these relationships define a clear design framework for developing ursane-type antimicrobial agents with optimised activity and physicochemical profiles.

## 4. Conclusions

This review has consolidated recent advances in the design and biological evaluation of triterpenoid-based hybrid molecules, with a focus on BA, OA, and UA. Across these scaffolds, structural modification particularly at the C–28 position has consistently emerged as the most effective strategy for enhancing antimicrobial potency. Functionalization with amides, triazoles, dithiocarbamates, and cationic groups has yielded hybrids with improved activity against both Gram-positive and Gram-negative bacteria, as well as fungal pathogens such as *C. albicans* and *C. neoformans*. BA-based hybrids show the most rational SAR trends, with C–28 conjugation driving potency and C–3 or A–ring tailoring refining solubility, selectivity, and biofilm penetration. OA hybrids have demonstrated strong antibacterial and antifungal activity, especially when linked to known antibiotics or redox-active fragments. In contrast, UA-based hybrids remain underexplored in antifungal applications, though recent findings suggest promising avenues for future development. Cationic modifications, such as triphenylphosphonium and pyridinium salts, have proven effective against encapsulated yeasts and Gram-positive bacteria, while sulfur-rich linkers and halogenated triazoles offer dual-spectrum activity and enhanced membrane targeting. Glycosylation and amino acid conjugation further improve aqueous solubility and cellular uptake, contributing to broader biological reach. Collectively, these findings establish a rational framework for converting triterpenoid scaffolds into lead-like antimicrobial agents through targeted hybridization.

When the three scaffolds are considered comparatively, several shared and scaffold-specific trends emerge that help define the most promising hybrid design strategies. Across BA, OA, and UA, modification at the C–28 carboxyl group consistently yields the most pronounced improvements in antimicrobial activity, particularly through amide formation, heterocyclic conjugation (e.g., 1,2,3–triazoles), and the introduction of cationic or sulfur-containing moieties. BA-based hybrids display the most clearly defined structure–activity relationships, with repeated reports of enhanced potency against Gram-positive bacteria and fungi following C–28 derivatization, suggesting a robust and predictable scaffold for rational optimisation. OA-based hybrids, while similarly responsive to C–28 modification, appear especially versatile when linked to pharmacologically active fragments or antibiotic-inspired motifs, often resulting in broader antimicrobial spectra. In contrast, UA-based hybrids remain comparatively underexplored, particularly in antifungal and in vivo contexts, despite showing promising activity trends that parallel those of OA. Collectively, these observations suggest that C–28-focused hybridization strategies represent a generalizable design principle across pentacyclic triterpenoids, while scaffold-specific features influence potency, spectrum, and translational potential.

Despite the promising biological outcomes, several limitations persist in the current literature. Many studies rely heavily on MIC data without reporting minimum fungicidal concentrations, cytotoxicity profiles, or in vivo efficacy, which restricts translational relevance. There is also a lack of standardised testing protocols and mechanistic assays, making cross-study comparisons difficult. Although the majority of reported studies remain limited to in vitro antimicrobial evaluation, a small number of triterpenoid-based hybrids have demonstrated translational potential in vivo. Notably, Blanco–Cabra et al. [36] reported the efficacy of a triterpenoid hybrid in an in vivo biofilm infection model, providing proof of concept that such compounds can retain activity under physiological conditions. In addition, recent work has described ursolic acid-derived hybrids with in vivo activity against MRSA, highlighting the feasibility of advancing these scaffolds beyond preliminary screening. These examples, while still scarce, underscore the importance of expanding in vivo and pharmacological studies to support the clinical relevance of triterpenoid-based hybrid antimicrobials.

Future research should prioritise comprehensive biological profiling, including ADME properties, cytotoxicity screening, and mechanistic validation, to ensure that promising hybrids can progress toward clinical application. The limited number of UA-based antifungal hybrids highlights a clear gap in scaffold diversification, suggesting that further exploration of this triterpenoid could yield valuable therapeutic candidates. Ultimately, the rational development of triterpenoid-based hybrids requires an integrated approach combining SAR-guided synthesis, mechanistic insight, safety evaluation, and translational testing. As antimicrobial resistance continues to rise, these natural scaffolds, when thoughtfully modified, offer a versatile platform with significant potential for next-generation drug discovery.

## Figures and Tables

**Table 1:** Betulinic acid-based hybrids with tested bacterial strains, SAR, and mode of action.

Hybrids	Strains	SAR	Mode of Action	Ref.
**4**	*E. coli*, *K. pneumoniae*	Triazole at C–28 enhances Gram-negative potency; CuAAC yields regioselective, membrane-active hybrids	Membrane disruption and enzyme inhibition via triazole-mediated interactions	[27]
**5**	*S. mutans*, *B. cereus*	Cystamine amide at C–28 introduces redox-active disulfide; improves solubility and bacterial uptake	Redox stress induction and thiol–disulfide exchange disrupting bacterial metabolism	[28]
**6**	*S. pyogenes*, *S. aureus*	Glycine tail at C–28 and indole fusion at C–2/C–3 improves Gram-positive activity and biofilm penetration	Dual mechanism: π–surface modulation and enhanced membrane interaction	[11]
**7**	*E. coli*, *S. aureus*	Dithiocarbamate at C–28 increases amphiphilicity and sulfur donor character	Synergistic membrane disruption and metabolic inhibition	[29]
**8a**–**d**, **9a**–**c**	*S. aureus*, *S. epidermidis*	Triphenylphosphonium (TPP^+^) conjugation at C–28 and/or C–3 introduces permanent positive charge; boosts Gram-positive selectivity	Electrostatic binding to bacterial membranes and enhanced uptake	[30]
**10**, **11**	*S. aureus*	Pyridinium and tetrahydropyridine salts at C–28 improve solubility and membrane affinity	Cationic charge facilitates membrane permeabilization and intracellular accumulation	[31]
**12**, **13**	*S. aureus*, *E. coli*	Triazole rings at C–28 with halogen substituents enhance dual-spectrum activity	Enzyme inhibition and membrane targeting via electron-withdrawing triazole motifs	[32]

**Table 2:** Betulinic acid-based hybrids with tested fungal strains, SAR, and mode of action.

Hybrids	Strains	SAR	Mode of Action	Ref.
**14**	*C. neoformans*, *C. gattii*	C–28 esterification with aromatic moiety enhances lipophilicity and biofilm penetration	Disrupts mature biofilms, inhibits capsule formation, and induces cell wall stress	[34]
**15**	*C. albicans*, *C. parapsilosis*	Indole fusion at C–2/C–3 and glycine tail at C–28 improves membrane affinity and solubility	Alters membrane integrity and inhibits ergosterol biosynthesis	[11]
**16**	*C. albicans*	Dithiocarbamate at C–28 introduces sulfur-rich donor character and amphiphilicity	Inhibits hyphal formation, synergizes with fluconazole, and causes rapid fungicidal action	[29]
**17**, **18**	*C. albicans*	Sulfur-based conjugates improve lipophilicity and redox potential	Disrupts membrane and induces oxidative stress	[29]
**13**, **19**–**22**	*C. albicans*	Triazole rings with halogen substituents enhance polarity and target binding	Inhibits fungal growth via membrane targeting and enzyme inhibition	[32]

**Table 3:** Oleanolic acid hybrids with tested bacterial strains, SAR, and mode of action.

Hybrids	Strains	SAR	Mode of Action	Ref.
23a–c	*L. monocytogenes*, *S. aureus*, *S. typhimurium*, *P. aeruginosa*	Phthalimidine–triazole linkers at C–28 enhance Gram-positive selectivity and improve lipophilicity	Disrupts bacterial membranes and inhibits metabolic enzymes	[12]
24a, 24b, 24f	*S. aureus*, *E. faecalis*, *B. cereus*, *E. coli*, *S. enterica*, *P. aeruginosa*	Piperazine ring at C–28 improves amphiphilicity and membrane interaction; morpholine and pyrrolidinone less effective	Enhances membrane permeability and disrupts bacterial respiration	[35]
25a, 25b	*E. cloacae*, *P. vulgaris*, *K. oxytoca*, *P. aeruginosa*, *P. mirabilis*, *K. pneumoniae*	Chlorophenyl–oxadiazole conjugation improves Gram-negative activity; OA core contributes moderate potency	Targets outer membrane and interferes with protein synthesis	[41]
26a, 26b	*X. oryzae*, *X. axonopodis*, *P. syringae*	Piperazine–sulfonamide at C–28 enhances electronic character and amphiphilicity	Disrupts bacterial membranes and inhibits growth of phytopathogens	[42]
27a–d	*E. faecalis*, *K. oxytoca*, *E. coli*, *S. aureus*, *E. cloacae*, *P. vulgaris*	4–aminoquinoline conjugation at C–28 improves broad-spectrum efficacy and enhances DNA interaction	DNA intercalation and inhibition of nucleic acid synthesis	[13]

**Table 4:** Oleanolic acid hybrids with tested antifungal strains, SAR, and mode of action.

Hybrids	Strains	SAR	Mode of Action	Ref.
**28a**–**c**, **29a**–**c**	*S. sclerotiorum*, *B. cinerea*, *R. solani*	Triazole conjugation at C–28 with electron-withdrawing groups (Cl, NO_2_) enhances antifungal potency	Disrupts fungal membranes and inhibits ergosterol biosynthesis	[43]
**30a**, **30b**	*C. albicans*, *C. glabrata*	Saponins from Camellia sinensis with glycosylation at C–3 and hydroxyl at C–23 improve potency	Membrane disruption and enhanced uptake	[44]
**31**	*C. albicans*	Terminal carboxyl group increases hydrophilicity and membrane interaction	Strengthens membrane binding and inhibits fungal respiration	[45]
**32a**–**c**	*G. graminis*, *V. mali*	Tertiary amide at C–28 improves amphiphilicity and electronic character	Inhibits mycelial growth and induces cell wall stress	[46]
**33a**–**d**	*C. albicans*, *C. neoformans*, *C. parapsilosis*	Glycosylation at C–3 and hydroxylation at C–23 enhance antifungal potency	Disrupts fungal membranes and inhibits biofilm formation	[47]

**Table 5:** Ursolic acid hybrids with tested bacterial strains, SAR, and mode of action.

Hybrids	Strains	SAR	Mode of Action	Ref.
**34a**, **34b**	*S. aureus*, *L. innocua*	Triazole fusion at C–28 reduces potency compared to native UA	Weak membrane interaction and poor uptake	[55]
**35**	*X. oryzae*, *X. axonopodis*	C–28 amide enhances ROS generation and membrane permeability	Induces oxidative stress and disrupts bacterial membranes	[56]
**36**, **37**	*S. aureus (including MRSA)*	Benzenesulfonamide–indole conjugation at C–28 improves Gram-positive potency	Strong membrane disruption and in vivo efficacy	[57]
**38a**, **38b**	*B. cereus*, *S. aureus*, *E. coli*, *S. typhimurium*	Esterification at C–3 improves lipophilicity and broad-spectrum activity	Enhances membrane permeability and metabolic inhibition	[58]
**39a**–**c**	*X. oryzae*, *X. axonopodis*	N-substituted amides at C–28 with cyclic amines improve potency and selectivity	ROS generation, membrane disruption, and enzyme inhibition	[59]

**Table 6:** Ursolic acid hybrids with tested fungal strains, SAR, and mode of action.

Hybrids	Strains	SAR	Mode of Action	Ref.
**40**, **41**	*C. albicans*	Arylidene–hydrazide hybrids at C–3 improve lipophilicity and membrane interaction	Inhibits fungal growth at lower concentrations than UA	[60]
**42**	*P. capsici*, *F. graminearum*	Esterification at C–3 enhances lipophilicity and membrane binding	Disrupts fungal membranes and improves uptake	[61]
**43**, **44**	*C. albicans*	C–3 esterification with alkyl/aryl groups improves hydrophobicity and membrane permeability	Disrupts ergosterol-rich membranes and promotes cytoplasmic accumulation	[58]
**45**	*C. albicans*	C–3 ester with bulky aromatic group enhances lipophilicity and target binding	Strong membrane disruption and improved antifungal potency	[58]

## Data Availability

No new data were created or analyzed in this study. Data sharing is not applicable to this article.

## Abbreviations

The following abbreviations are used in this manuscript:

ADME	Absorption, Distribution, Metabolism and Excretion
ATCC	American Type Culture Collection
BA	Betulinic acid
CAT	Catalase
CuAAC	Copper(I)-catalysed Azide-Alkyne Cycloaddition
DCC	Dicyclohexylcarbodiimide
DMAP	4–Dimethylaminopyridine
DTC	Dithiocarbamate
EDC	1–Ethyl–3–(3–dimethylaminopropyl)carbodiimide
EC_50_	Half–maximal effective concentration
HOBt	1–Hydroxybenzotriazole
HL–60	Human promyelocytic leukaemia (cell line)
MBC	Minimum Bactericidal Concentration
MFC	Minimum Fungicidal Concentration
MIC	Minimum Inhibitory Concentration
MRSA	Methicillin-resistant Staphylococcus aureus
mM	Millimolar
mg·L^−1^	Milligrams per litre
µM	Micromolar
µg/mL	Micrograms per millilitre
NSAIDs	Non-Steroidal Anti-Inflammatory Drugs
OA	Oleanolic acid
ROS	Reactive Oxygen Species
SOD	Superoxide Dismutase
SAR	Structure–Activity Relationship
UA	Ursolic acid
